# 675. Implementing Night Shift Dressing Changes to Improve Patient Participation in Rehabilitation: A Case Study

**DOI:** 10.1093/jbcr/irag033.556

**Published:** 2026-04-06

**Authors:** Raven Giles

**Affiliations:** John S. Dunn Burn Center, Houston, TX

## Abstract

**Introduction:**

Aggressive rehabilitation is essential in patients with large TBSA burns to prevent contractures and long-term functional deficits. Traditionally, dressing changes are performed during daytime hours, which often disrupts participation in physical and occupational therapy due to increased pain, fatigue, and medication-induced somnolence. These disruptions may also negatively impact mood, caloric intake, and circadian rhythm. This case study explores the impact of shifting dressing changes to the night shift, hypothesizing that this change would improve therapy participation and functional outcomes.

**Methods:**

A multidisciplinary protocol was developed by nursing, therapy, and burn physicians during daily rounds in response to frequent missed therapy sessions in a patient with a 40% TBSA burn. Once operative interventions were completed and conscious sedation was no longer required, physicians ordered dressing changes to occur during night shift. Concurrently, therapy sessions were extended, and nursing staff ensured the patient was out of bed by 9:00 a.m. and active until approximately 5:00 p.m. The patient was educated about the revised schedule and encouraged to engage in ADLs and functional mobility. Progress was monitored using the Activity Measure for Post Acute Care (AMPAC) scores, and wound healing was assessed through nursing and physician evaluations, supplemented by photographic documentation in the EMR.

**Results:**

Following the implementation of night shift dressing changes, the patient demonstrated significant improvement across multiple domains. AMPAC scores increased from 9 to 17 over eight weeks. Therapy participation increased from approximately 60 minutes to 3 hours per day. The patient exhibited gains in range of motion, strength, activity tolerance, balance, gait, and splint compliance. No therapy refusals occurred post-intervention. Patient-reported outcomes included improved mood and normalized sleep–wake cycles. Wound assessments revealed accelerated healing, with wounds nearly closed at the time of discharge to inpatient rehabilitation.

**Conclusions:**

Shifting dressing changes to the night shift enhanced burn rehabilitation by reducing pain during therapy hours, improving engagement, and accelerating recovery. This patient’s outcomes suggest that such scheduling changes may support functional gains, mood stabilization, and wound healing.

**Applicability of Research to Practice:**

This case supports a practical, low-cost intervention that can be widely implemented in burn care settings. Night shift dressing changes may reduce daytime pain and fatigue, improve therapy compliance, and enhance overall rehabilitation outcomes. Burn units should consider integrating this approach into routine care and evaluating its impact systematically. Broader adoption could lead to improved patient satisfaction, shorter hospital stays, and enhanced long-term function in patients with large TBSA burns.

**Funding for the study:**

N/A.

